# Biowaste-based hydroxyapatite for bone repair: synthesis from *Anadara granosa* and *Achatina fulica*: preliminary study

**DOI:** 10.1590/1807-3107bor-2026.vol40.014

**Published:** 2026-05-08

**Authors:** Silvia ANITASARI, Nataniel TANDIROGANG, Hendrik Setia BUDI, Yung-Kang SHEN, Sinar YANI, Yuniati YUNIATI

**Affiliations:** (a) Universitas Mulawarman, Faculty of Dentistry, Department of Dental Material and Devices, Samarinda, East Kalimantan, Indonesia.; (b) Universitas Mulawarman, Faculty of Medicine, Department of Medical Microbiology, Samarinda, East Kalimantan, Indonesia.; (c) Universitas Airlangga, Faculty of Dental Medicine, Department of Oral Biology, Surabaya, East Java, Indonesia.; (d) Taipei Medical University, College of Oral Medicine, School of Dental Technology, Taipei, Taiwan.; (e) Universitas Mulawarman, Faculty of Dentistry, Department of Oral Biology, Samarinda, East Kalimantan, Indonesia.

**Keywords:** Durapatite, Biocompatble Materials, Cell Survival, Osteoblasts, Bone Regeneration

## Abstract

Bone regeneration is a critical area in tissue engineering because of the increasing incidence of bone defects resulting from trauma, degenerative diseases, and congenital disorders. The focus of this study is the synthesis of hydroxyapatite (HAp) from two natural calcium-rich biowastes: *Anadara granosa* (blood cockle) and *Achatina fulica* (snail). The shells were calcined at 900 °C to form calcium oxide (CaO) and then converted into HAp via the wet precipitation method using a Ca/P molar ratio of 1.67. The synthesized HAp powders were evaluated for their chemical properties and biological performance. Fourier transform infrared (FTIR) spectroscopy confirmed the presence of phosphate and hydroxyl functional groups, and among the samples, An 100 showed the highest crystallinity. MC3T3-E1 cell viability was assessed using a 3-(4,5-dimethylthiazol-2-yl)- 2,5-diphenyltetrazolium bromide (MTT) assay at 24, 48, and 72 hours. At 72 hours, An 100, An 75, and An 50 maintained a viability above 70%, indicating good biocompatibility. In contrast, An 25 and Ac 100 exhibited significant cytotoxicity (p < 0.05). Only the noncytotoxic concentrations were used for the in vitro scratch wound-healing assay, where An 100 demonstrated the most rapid wound closure, indicating increased osteoblast migration. Furthermore, in this study, the elemental composition and structural integrity of Hap was analyzed to understand the factors affecting its stability and performance in biological environments. These findings suggest that naturally derived HAp is a promising, sustainable, and effective biomaterial for bone tissue engineering and has favorable effects on cell viability and migration.

## Introduction

Bone regeneration has become a pivotal focus in tissue engineering because of the increasing prevalence of bone defects arising from trauma, degenerative diseases, infections, and congenital abnormalities. The development of viable materials that closely mimic the natural structure and composition of bone is essential for increasing osteogenesis and improving the efficacy of regenerative treatments. Among the various biomaterials explored, hydroxyapatite (HAp) is the gold standard because of its chemical similarity to the mineral component of bone, excellent viability, and bioactive properties. HAp provides an optimal microenvironment for osteoblast adhesion, proliferation, and differentiation, making it a highly favorable candidate for bone tissue engineering and regeneration.^1,2^

Conventional synthetic methods for HAp production typically involve chemical precursors and energy-intensive processes. However, recent advancements in biomaterial science have led to a paradigm shift toward sustainable approaches, with a focus on natural calcium-rich sources. Using such natural resources not only reduces environmental waste but also offers an eco-friendly and cost-effective alternative for biomaterial production.^3^ One promising avenue comprises deriving HAp from biomineralized structures, such as the shells of marine and terrestrial mollusks, which primarily consist of calcium carbonate (CaCO3). These natural precursors undergo controlled thermal and chemical treatments to convert calcium carbonate, resulting in biomaterials with unique physicochemical characteristics that may enhance their biological performance in tissue engineering applications.^4^

In the current study, the synthesis of HAp was carried out from 2 distinct natural sources, namely, *Anadara granosa* (blood cockle) and *Achatina fulica* (snail). A. granosa is a marine bivalve mollusk widely distributed in coastal regions that possesses a robust calcium carbonate shell, making it a promising candidate for HAp synthesis.^4^ *A. fulica* is a terrestrial gastropod and is also recognized for its large, calcium-rich shell structure, which is often regarded as waste.^5^ Repurposing these shells for HAp synthesis presents an opportunity to develop sustainable biomaterials while mitigating environmental pollution. By subjecting the shells to precise thermal and chemical processing, their calcium carbonate content can be transformed into products suitable for biomedical applications, including bone grafts, scaffolds, and coatings for orthopedic and dental implants.^3,6^

To assess the biological performance of synthesized HAp, MC3T3-E1 preosteoblast cells, which have been used as an in vitro model for osteoblast function and bone regeneration, were used in this study. Several critical parameters, including cell viability, attachment, proliferation, and migration, were evaluated to determine the viability and osteogenic potential of HAp derived from A. granosa and *A. fulica*.^7^ A particular area of interest is the influence of HAp composition on cell migration, as this dynamic process plays a fundamental role in tissue regeneration and bone healing. Understanding how biomaterials support cellular activity provides crucial insights into their potential applications in regenerative medicine.^8^ Therefore, the aim of this study is to investigate the synthesis of HAp from 2 natural calcium-rich sources, namely, A. granosa (blood cockle) and *A. fulica* (snail). The key chemical elements influencing HAp stability and performance in biological environments were also assessed. Assessments were carried out by analyzing the elemental composition and structural integrity of HAp, as well as its role in facilitating cell viability and migration during the in vitro wound-healing process.

## Methods

### Preparation of *A. granosa* and *A. fulica* shells

This study was approved by the Faculty of Medicine, Mulawarman University (No. 192/KEPK-FK/VII/2024). Shells of A. granosa and *A. fulica* were sourced from Samarinda, Indonesia. The preparation process began with thoroughly washing the shells with water to remove any dirt or debris. After cleaning, the shells were rinsed and left to dry under direct sunlight for an entire day. The next step involved further drying in a drying cabinet at 50°C for 48 hours to eliminate residual moisture. The resulting shell was then stored in a plastic container at room temperature until needed.^3^

### Preparation of calcium oxide (CaO)

The shells of A. granosa and *A. fulica* were subjected to calcination in a furnace (Barnstead, USA) at 900 °C for durations of 6 hours and 3 days, respectively. High-temperature processes facilitated the decomposition of calcium carbonate into CaO. The CaO powder obtained from crushed A. granosa and *A. fulica* shells was then stored in a dry container until analysis.^3^

### HAp synthesis

HAp powder was synthesized using the precipitation method, which involved mixing a phosphate precursor solution with a calcium precursor solution at a molar Ca/P ratio of 1.67. The calcium precursor was obtained from Ca(OH)_2_, which was produced by reacting calcined CaO with distilled water. A 0.5 M Ca(OH)_2_ suspension was prepared by dissolving 3.7046 grams of shell powder in 100 mL of sterile distilled water in a volumetric flask. The suspension was stirred using a magnetic stirrer at 500 rpm. Afterward, 50 mL of 0.3 M H_3_PO_4_ solution was slowly added while stirring was maintained at 300 rpm for 6 hours until the mixture became homogeneous. The mixture was then left to stand at room temperature for 1 hour before 1 M NaOH was added until the pH reached 10. The stirring process was continued for another 30 minutes at 300 rpm, followed by an aging period of 24 hours at room temperature.

The resulting precipitate was filtered using Whatman filter paper, washed with distilled water, and dried in an oven at 110°C for 3 hours. The heating process was carried out at 800 °C for 5 hours at a heating rate of 5°C per minute to obtain high-purity HAp crystals. Additionally, theoretical calculations of the HAp yield were conducted using raw materials derived from A. granosa and *A. fulica*, the results of which are presented in Table.

**Table:** Theoretical calculation of the hydroxyapatite (HAp) yield from *A. granosa* and *A. fulica*

No	Stage	Description
1	Starting material	•Shell powder 3.7046 g (assumed to be primarily CaCO_3_) •Molar mass of CaCO_3_ ≈ 100.09 g/mol Moles of ${\mathrm{CaCO}}_{3}=\frac{3.7046g}{100.09g/\mathrm{mol}}\approx 0.037\mathrm{mol}$ Upon calcination and hydration: CaCo_3_ ∆ CaO ^+H^_2_^O^ Ca(OH)_2_ Each mole of CaCO_3_ gives 1 mole of Ca(OH)_2_ → provides 1 mole of Ca^2+^ So, available Ca^2+^ = 0.037 mol
2	Molar matio (Ca/P=1.67)	From this ratio: For $1.67\mathrm{mol}\mathrm{Ca}\to 1\mathrm{mol}P\to 0.037\mathrm{mol}\mathrm{Ca}\to \frac{0.037}{1.67}\approx 0.02216\mathrm{mol}P$ Since phosphate source is H_3_PO_4_ (orthophosphoric acid), assuming complete reaction, this matches the addition of: • $0.3MH_{3}{\mathrm{PO}}_{4}\times 50\mathrm{mL}=0.015\mathrm{mol}H_{3}\mathrm{PO}4\to \text{ contains }0.015\mathrm{mol}P$ , Thus, H_3_PO_4_ is the limiting reagent.
3	Balanced reaction (for HAp)	$10{\mathrm{Ca}}^{2+}+6{\mathrm{PO}}_{4}^{3-}+2{\mathrm{OH}}^{-}\to {\mathrm{Ca}}_{10}(\mathrm{PO}4{)}_{6}(\mathrm{OH}{)}_{2}$ • 6 mol P → 1 mol HAp • $0.015\mathrm{mol}P\to \frac{0.015}{6}=0.0025\mathrm{mol}\mathrm{HAp}$
4	Molar mass of HAp	• ${\mathrm{Ca}}_{10}{\left({\mathrm{PO}}_{4}\right)}_{6}(\mathrm{OH}{)}_{2}\approx 1004g/\mathrm{mol}$ • Mass of HAp =0.0025×1004=2.51 grams
5	Result	Theoretical Yield: ~2.51 grams of hydroxyapatite

### Functional group analysis

HAp powder was placed in front of an FTIR crystal (Shimadzu, Japan). The infrared light, which served as the light source, was divided into 2 beams. One of the beams passed through the sample, and the other passed through a reference. Both beams sequentially passed through the FTIR crystal, and the signal was captured by a detector. It was then converted into an electrical signal and recorded by a recorder. The wavelength range used for measurement was 500–4,000 cm^–1^ (wavenumber).^9^

### Cell culture

MC3T3-E1 cells were cultured in culture plates supplemented with DMEM (Sigma–Aldrich, St. Louis, USA) with 10% fetal bovine serum (FBS) (Sigma–Aldrich, St. Louis, USA) and 1% penicillin and then incubated in a 5% CO_2_ atmosphere. The culture medium was replaced every 2 to 3 days. Once the cells reached 80% confluency, the samples were detached from the plate surface using trypsin-EDTA (Sigma–Aldrich, St. Louis, USA) before being subcultured.^10,11^

### Cell viability analysis

In the current study, cell viability was evaluated using a 3-(4,5-dimethylthiazol-2-yl)-2,5-diphenyltetrazolium bromide (MTT) assay. HAp powder derived from A. granosa and *A. fulica* shells was sterilized by ultraviolet (UV) exposure for 60 minutes. A total of 0.02 mg of HAp was dissolved in 10 mL of DMEM and incubated in a shaker water bath at 100 rpm and 37°C for 24 hours. The HAp-containing medium was then filtered using Whatman filter paper, and the filtrate was used as an extract for the viability test. MC3T3-E1 cells were cultured in 96-well plates and exposed to various concentrations of HAp extract, namely, 100% A. granosa (An 100), 100% *A. fulica* (Ac 100), and mixtures at ratios of 75:25 (An 75), 50:50 (An 50), and 25:75 (An 25). An 100 consisted of 100% HAp derived from A. granosa. An 75 consisted of 75% HAp from A. granosa and 25% HAp from *A. fulica*. An 50 contained 50% HAp from A. granosa and 50% HAp from *A. fulica*. An 25 consisted of 25% HAp from A. granosa and 75% HAp from *A. fulica*. Ac 100 represented a composition of 100% HAP derived from *A. fulica*. The control groups consisted of MC3T3-E1 cells cultured in DMEM alone.

After 24, 48, and 72 hours of incubation in a 5% CO_2_ atmosphere, 50 µL of MTT solution was added to each well and incubated for another 3 to 4 hours at 37°C. The reaction products were then analyzed using an ELISA reader at a wavelength of 450 nm. The optical density (OD) of the cells was measured to evaluate cell viability using the following formula^12,13^:


$$\text{ Cell viability }(\%)=\frac{\text{ OD sample }}{\text{ OD MC3T3 }-\text{ E1 Cells }}\times 100\%$$ (1)

### Scratch wound assay (cell migration)

Only noncytotoxic concentrations were selected for the cell migration assay. Mouse osteoblasts (MC3T3-E1) were cultured in 12-well plates at a concentration of 3x10^4^ cells/mL (1 mL per well) and incubated until they reached 90% confluency within 24 hours. Once the medium was removed, a 200-mL pipette tip was used to create a scratch on the cell layer. The wells were then rinsed with phosphate-buffered saline (PBS) to remove any detached cells or debris. HAp extract was subsequently added to each well to serve as a chemoattractant.^14^

The cells were observed using an Olympus IX73 inverted microscope (Olympus, Japan) at 100x magnification. Images of the area between the wound edges were captured at 0, 12, 24, and 48 hours and analyzed using ImageJ software. The extent of wound closure was calculated using the following formula:


$$\text{ Wound Closure (\%) }\frac{A_{t=0h}-A_{t=\Delta h}}{A_{t=0h}}\times 100\%$$ (2)

**Figure 1:**
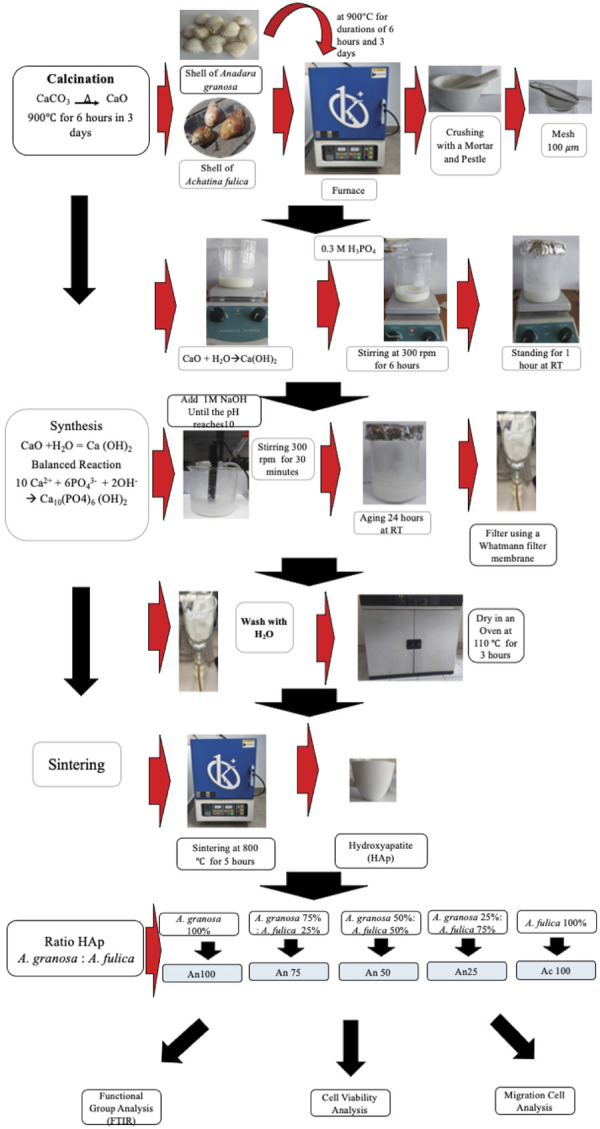
Schematic diagram outlining the study design for the synthesis and characterization of hydroxyapatite from *A. granosa* and *A. fulica* shells.

In this formula, A_t_= 0 h represents the initial wound area immediately after scratching, while A_t_=h denotes the wound area after h hours.^14,15^ For clarity, a schematic diagram outlining the study design for the synthesis of hydroxyapatite (HAp) is presented in Figure 1.

### Statistical analysis

For each experiment, all the conditions were evaluated in triplicate, and the results are presented as the means and standard errors (SEs). Statistical analysis was conducted using Origin Lab 2004 software, and one-way ANOVA followed by Tukey’s test was used to assess differences between groups. p<0.05 was considered statistically significant.^16^

## Results

**Figure 2:**
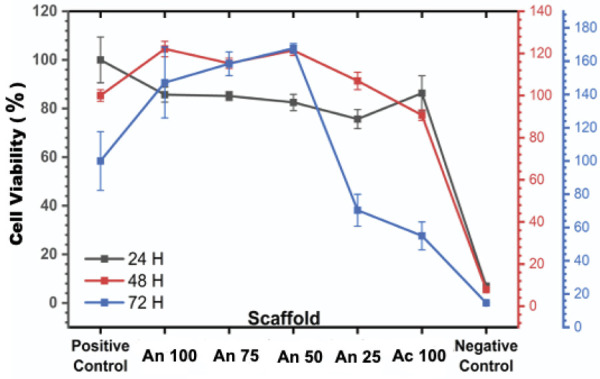
Effects of HAp derived from *A. granosa* and *A. fulica* on the viability of MC3T3-E1-osteoblasts.

At the 24-hour mark, all the HAp samples were viable with respect to MC3T3-E1 cells, which exhibited varying levels of viability. The highest percentage of viable cells was recorded for Ac 100 (86.3%), followed by An100, An75, An50, and An 25. Statistical analysis revealed no significant differences (p>0.05) among the groups at this time point, as shown in Figure 2.

After 48 hours, an increase in cell viability was observed across all samples. The highest viability percentages were recorded for An 100, An 75, An 50, An 25, and Ac 100. However, a statistically significant difference (p < 0.05) was observed between the Ac 100 group and the An100, An 75, An 50, and An 25 groups, with the Ac 100 group showing lower viability, as shown in Figure 2.

During the third day (72 hours), samples of An 100, An 75, and An 50 continued to exhibit increasing viability. However, a significant decrease in viability was observed in the samples of An 25 and Ac 100, with the percentage of viable cells decreasing to 70.4% for An 25 and 54.9% for Ac 100. Statistical analysis confirmed a significant difference (p < 0.05) between these groups. These results suggested that An 25 and Ac 100 did not meet the criteria for viability, as a material was considered viable only if it maintained a cell viability percentage above 70% for 3 consecutive days, as shown in Figure 2.^16^

**Figure 3:**
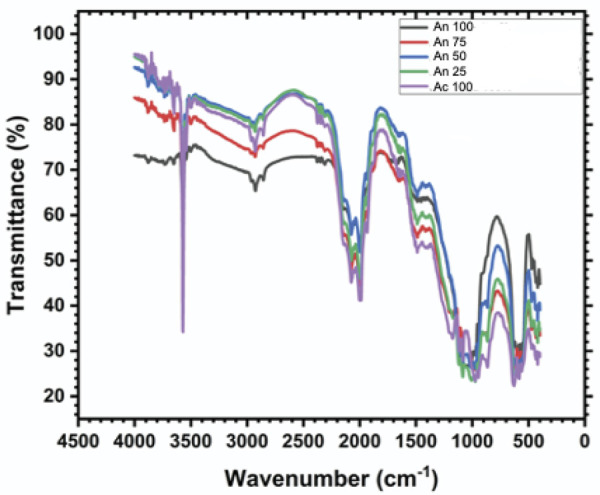
FTIR spectra of HAp derived from *A. granosa* and *A. fulica.*

Peaks at ~960 cm^-1^ (symmetric vibration), 1,030–1,090 cm^-1^ (asymmetric vibration), and 560–600 cm^-1^ (bending vibration) corresponded to phosphate functional groups in the HAp structure. Compared with An50, An75, An25, and Ac 100, HAp An100 had sharper peaks. The sharpness and intensity of these peaks reflected the degree of crystallinity, as shown in Figure 3.

The 3570 cm^-1^ peak corresponded to the stretching vibration of the hydroxyl group. The 630 cm^-1^ peak corresponded to the bending vibration of the hydroxyl group. Weak peaks were observed for Ac 100, An 25, An 50, An 75, and An100. Weak or missing peaks suggested a reduction in hydroxylation, as fewer hydroxyl groups were present in the HAp structure, as shown in Figure 3.

Peaks at 1,410–1,450 cm^-1^ and 870 cm^-1^ indicated the substitution of carbonate ions into the HAp structure. HAp derived from natural sources had a relatively high carbonate content. The intensity of these carbonates was greater in HAp from A. granosa than in that from *A. fulica* in the following order: An 100, An 50, An 75, An 25, and Ac 100, as shown in Figure 3.

**Figure 4:**
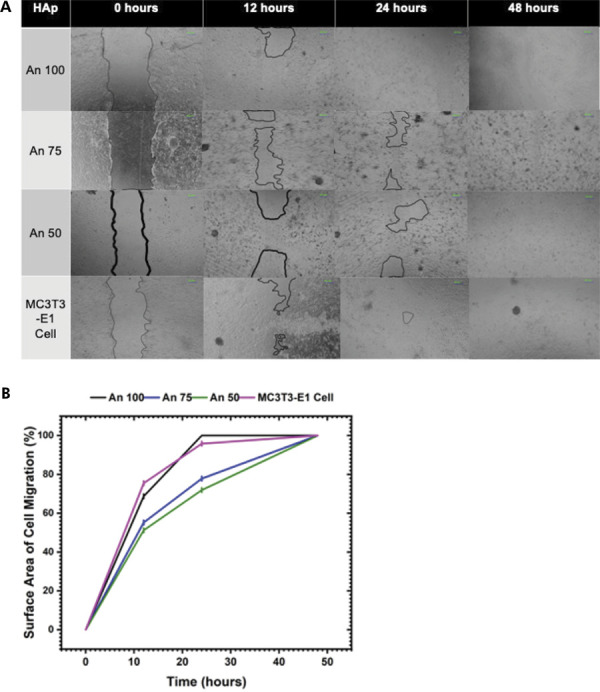
A. Cell migration mediated by HAp derived from *A. granosa* and *A. fulica.* B. Percentage of surface area covered by cell migration mediated by HAp derived from *A. granosa* and *A. fulica.*

In the scratch assay, the results at 12 hours revealed a statistically significant difference (p < 0.05) in cell-covered surface area across all groups, with the highest coverage occurring in the An 100 group, followed by the An 75 and An 50 groups. At 24 hours, a significant difference (p < 0.05) was also observed among all groups, with the leading groups being An 100, MC3T3-E1 cells, An 75, and An 50, as shown in Figure 4A. By 48 hours, all the scratched areas were fully covered (100%), as shown in Figure 4B.

## Discussion

The reduced viability observed for Ac 100 and An 25 was likely due to their lower crystallinity and reduced hydroxylation levels,^17^ which are crucial factors in promoting cell attachment and growth. The differences in these properties among the An 100, An 75, An 50, An 25, and Ac 100 samples helped explain why some of the samples performed better biologically.

Crystallinity refers to the extent to which the crystal structure of a material is well organized. In this case, the An 100 sample showed the highest crystallinity, as evidenced by the sharp and intense peaks detected through FTIR analysis. These peaks, observed at ~960 cm^-1^, 1,030–1,090 cm^-1^, and 560–600 cm^-1^, corresponded to the phosphate groups in HAp. The clearer and more defined these peaks were, the more ordered the HAp structure was.^17,18^ An 100 samples, having the most crystalline structure, provided a more stable and organized surface that supported better cell adhesion and proliferation. A more crystalline structure also showed slower degradation in biological environments, allowing the scaffold to maintain its shape and function longer, as shown in Figure 3.^19^

The Ac 100 and An 25 samples had broader and weaker peaks, indicating lower crystallinity and a more disordered structure.^20^ This disorganization could make the surface rougher and less predictable, which reduced the ability of cells to attach and grow. Lower crystallinity could also lead to faster degradation, making it difficult for cells to adhere to the scaffold consistently.^13^ The likely cause of the lower crystallinity in Ac 100 and An 25 included inadequate synthesis conditions, such as rapid cooling, insufficient reaction times, or improper thermal treatment, which prevented the formation of a well-organized crystal structure.^21^

Hydroxyl groups (-OH) in HAp were crucial for increasing viability. OH groups improved hydrophilicity, which is essential for cell adhesion. FTIR analysis revealed that the Ac 100, An 25, An 50, and An 75 samples had relatively weak peaks at 3570 cm^-1^ and 630 cm^-1^, which corresponded to the stretching and bending vibrations of the hydroxyl group. By contrast, the peak intensity of the An100 samples was relatively strong, indicating higher hydroxylation levels.

Reduced hydroxylation, as seen in the Ac 100 and An 25 samples, could negatively affect viability in several ways. For example, less surface interaction with biological fluids, weaker bonding with tissues, and slower formation of a bone-like layer can occur. The lower hydroxylation levels in Ac 100 and An 25 may be due to high-temperature treatments that caused thermal dehydration or chemical substitutions during synthesis, where other ions replaced the hydroxyl groups.^22,23^

The reduced viability observed in the Ac 100 sample may also be influenced by the presence of carbonate substitution in the HAp structure. Peaks at 1,410–1,450 cm^-1^ and 870 cm^-1^ were attributed to the incorporation of carbonate ions. HAp derived from A. granosa presented a higher carbonate content than that derived from *A. fulica*, with the intensity order of carbonate peaks being An 100> An 50> An 75> An 25> Ac 100. The higher carbonate content in An 100 may increase bioactivity and promote cell proliferation, whereas lower carbonate levels in Ac 100 and An 25 could result in decreased cellular interactions.^24^

The differences in carbonate content and its influence on viability among the An 100, An 75, An 50, An 25, and Ac 100 samples could be explained by variations in the source material and synthesis conditions. The biological origin of HAp plays a significant role in its composition and structural properties. HAp derived from A. granosa contained a higher proportion of aragonite, a calcium carbonate polymorph that was more easily converted into HAp through carbonate substitution. This explained why the An 100 sample, derived from A. granosa, exhibited the highest carbonate content. Moreover, *A. fulica* shells primarily contained calcite, a more stable form of calcium carbonate, which was less prone to carbonate substitution, resulting in lower carbonate levels in the Ac 100 sample.^25,26^

The synthesis process influenced carbonate incorporation, as factors such as temperature, pH, and reaction time played crucial roles in determining the extent of carbonate substitution. One hundred samples likely underwent synthesis conditions that favored carbonate retention, such as lower reaction temperatures (≤ 800°C) and controlled pH (8–10), both of which promoted B-type carbonate substitution where carbonate ions replaced phosphate groups in the HAp structure.^27^ The Ac 100 sample, which exhibited a lower carbonate content, may have been subjected to higher calcination temperatures (> 900°C), leading to decarbonation and the loss of carbonate groups. Similarly, the An 25 sample may have experienced shorter reaction times or insufficient carbonate availability during synthesis, limiting its carbonate incorporation.^28^

The degree of carbonate substitution affected the crystallinity, surface chemistry, and bioactivity of HAp. Due to their higher carbonate content, the An 100 samples showed a more disordered crystal structure (lower crystallinity), which increased surface roughness and increased cell attachment and proliferation. In addition, higher carbonate levels promoted ionic exchange, improving the osteoconductive properties of the material. The lower carbonate content in the Ac 100 and An 25 samples resulted in increased crystallinity, which reduced the surface area and hydrophilicity, thereby limiting cellular interactions and decreasing viability.^27,28^

This variation in carbonate substitution was biologically significant because carbonate-substituted HAp closely mimicked the natural mineral phase of bone, which contained 3–8% carbonate. This similarity increased cellular recognition and promoted better integration with biological tissues. The higher carbonate content in the An 100 samples facilitated stronger biological interactions and better cell proliferation, whereas the lower carbonate levels in the Ac 100 and An 25 samples reduced their capacity to support cell adhesion and growth. Therefore, the differences in carbonate content and viability among the samples could be attributed to the combined effects of the mineral composition of the source material and the specific conditions under which the HAp was synthesized.^13,27^

Understanding these mechanisms is crucial for optimizing biomaterial properties to improve viability and facilitate tissue regeneration. Future studies should focus on increasing hydroxylation and controlling carbonate incorporation to reduce oxidative stress and improve cell–matrix interactions.^29,30^ However, in this study, a commercially available product was not included as a positive control, as hydroxyapatite derived from A. granosa and *A. fulica* shells is not available on the market. This is acknowledged as a limitation of this study, as it prevented direct comparison with commercially distributed products.

## Conclusion

In conclusion, the combined viability, FTIR, spectroscopic, and scratch assay data revealed that An 100 consistently exhibited superior viability, crystallinity, and cellular response. The improved biological performance of An 100 could be attributed to its higher crystallinity, greater hydroxylation, and increased carbonate content, which collectively increased cell attachment, proliferation, and migration. Moreover, the Ac 100 and An 25 samples showed compromised viability and structural integrity, as reflected by reduced cell viability and weaker functional group peaks. These results highlight the crucial role of material composition and structural properties in determining the biological performance of HAp for biomedical applications.

## Data Availability

The authors declare that all data generated or analyzed during this study are included in this published article.
